# Systematic Review Suggests Nutraceuticals Containing Vitamin B2 Could Provide an Alternative Treatment for Paediatric Migraines

**DOI:** 10.1111/apa.70157

**Published:** 2025-05-24

**Authors:** Elisa Martello, Fatimah Aiyelabegan, Jemma Orr, Emma Wilson, Joanne R. Morling, Maria Kinali, Titus Joseph, Dirontsho Koboto, Surakshya Dhungana, Prudence Ikechukwu, Princella Seripenah, Heidi Emery, James Stewart‐Evans, Jo Leonardi‐Bee

**Affiliations:** ^1^ The Nottingham Centre for Public Health and Epidemiology, School of Medicine University of Nottingham Nottingham UK; ^2^ Centre for Evidence Based Healthcare, School of Medicine University of Nottingham Nottingham UK; ^3^ NIHR Nottingham Biomedical Research Centre, Nottingham University Hospitals NHS Trust University of Nottingham Nottingham UK; ^4^ Department of Brain Sciences Imperial College London UK; ^5^ Department of Paediatric Neurology Portland Hospital HCA International London UK

**Keywords:** migraine, nutraceuticals, riboflavin, systematic review, vitamin

## Abstract

**Aim:**

The aim of this review is to systematically summarise the evidence on the effectiveness of riboflavin (vitamin B2) or supplements containing riboflavin in preventing paediatric migraines.

**Methods:**

This systematic review followed the Joanna Briggs Institute methodology and is reported to adhere to the Preferred Reporting Items for Systematic Reviews and Meta‐Analyses guidelines. We searched multiple electronic databases on 25 October 2023 to identify potentially relevant studies. Studies were screened, critically appraised and data extracted by two reviewers. Data underwent a narrative synthesis using vote counting based on direction of effect and was presented using harvest plots.

**Results:**

Seventeen studies of varying quality were included in the review. Riboflavin or its combination with other natural ingredients was significantly effective in reducing migraine frequency (7/10 comparisons), migraine days (3/4 studies) and use of analgesics (3/4 studies). Mixed findings were found on headache duration in hours (3/5 studies), pain intensity (4/6 studies) and disability (3/5 studies). No negative outcomes and few side effects were reported (five studies).

**Conclusion:**

Our findings suggest that using riboflavin at different dosages and treatment lengths offers a promising alternative to common medications for paediatric migraine prevention. Future research should explore mechanisms of action, optimal dosing and treatment duration using robust evidence.

## Summary

- Nutraceuticals have emerged as preventative treatments for migraine, but their effectiveness in paediatric migraine remains unclear.
- This systematic review investigated the effectiveness of riboflavin, or vitamin B2, in managing paediatric migraine, suggesting that riboflavin alone or in combination with natural ingredients can be effective in preventing paediatric migraines.
- The evidence is not yet robust, necessitating further high‐quality studies.

**Abbreviations**
CoQ10: coenzyme Q10
JBI: Joanna Briggs Institute
NA: not applicable
PedMIDAS: Paediatric Migraine Disability Assessment Scale
PRISMA: Preferred Reporting Items for Systematic Reviews and Meta‐Analyses
PROSPERO: International Prospective Register of Systematic Reviews
RCT: randomised controlled trial

## 1 Introduction

Migraines are common and often chronic, debilitating neurological disorders. They are mainly characterised by recurrent, severe headaches and can be accompanied by other symptoms such as nausea, vomiting and sensitivity to light, sound and visual aura [1]. Migraines can affect individuals of all age groups and are frequent among approximately 10% of children compared to 6% of men and 18% of women. In general, after puberty, girls have a threefold increase in the risk of migraine compared to boys [2]. Headaches and migraines contribute to a substantial burden on Paediatric Emergency Departments, from infancy through adolescence [3]. These conditions significantly impact children's and young people's quality of life by influencing academic, social and emotional development [4]. Different types of migraine include migraine with or without aura, chronic migraine, hemiplegic migraine, basilar, peri/catamenial migraine, silent migraine, retinal migraine and status migrainosus. Periodic syndromes that may be associated with migraine include abdominal migraine, benign paroxysmal torticollis, benign paroxysmal vertigo, recurrent gastrointestinal disturbance and cyclical vomiting syndrome. Finding effective and safe preventative treatment options for paediatric migraine patients is crucial. Effective treatment can significantly decrease migraine frequency, duration and severity, improve medication responses during acute episodes and everyday functioning and minimise disability [5, 6]. Preventative medications are prescribed to children and adolescents with significant migraine frequency or severity that causes substantial migraine‐related disability. Such medications include propranolol, topiramate, amitriptyline, nortriptyline, flunarizine, cyproheptadine, gabapentin, pregabalin and verapamil [7, 8]. However, the levels of evidence for their effectiveness are varied, and their use is limited by the potential for adverse effects, which in turn impacts patient choices and compliance. The American Academy of Neurology has published guidelines on preventive treatment for children and adolescents with migraine, which focuses on medications with or without cognitive behavioural therapy [8, 9].

Nutrition plays a role in cerebral metabolism and function. Proteins, amino acids, carbohydrates, vitamins and minerals enter the brain through the blood–brain barrier. Nutraceuticals have gained attention in recent years as options for disease treatment and health maintenance among children and adolescents, primarily due to their minimal side effects. Commonly chosen nutraceuticals, given alone or in combination and used at different dosages for preventing migraine in children and adolescents, are magnesium, coenzyme Q10 (CoQ10), riboflavin, butterbur, melatonin, Ginkgolide B, feverfew and some probiotics [10, 11, 12]. Given the limited scientific evidence supporting the efficacy of drugs for migraine prophylaxis in children and adolescents, the use of nutraceuticals becomes even more relevant. These alternatives offer a promising prophylactic option, particularly because they are generally well tolerated and have minimal side effects.

Riboflavin, also known as vitamin B2, is an essential water‐soluble vitamin found in animal‐derived foods such as meat, dairy products, eggs and plant‐based sources such as spinach, almonds and quinoa. Riboflavin plays a crucial role in various metabolic processes [13, 14], has an important function in mitochondrial energy production and is involved in cell protection from oxidative stress and neuroinflammation [15, 16]. For children, the average requirements for riboflavin are derived by extrapolating data from adults and applying corrections according to age and weight. For children of both sexes aged 1–17 years, the average requirements range between 0.5 and 1.4 mg/day [17] as reported by the European Food Safety Authority (2017). Toxic levels of riboflavin have not been observed from food sources or supplements since the gut can only absorb a limited amount of riboflavin at a time, with any excess being quickly excreted in the urine [14]. Although the exact aetiology of migraine is unknown [9], studies suggest that mitochondrial dysfunction, oxidative stress and neuroinflammation may be the main causes [15, 16]. Previous research in adults has indicated that riboflavin supplementation is an effective preventive treatment for migraine when taken mainly at dosages between 100 and 400 mg/day for 3 months [9]. Therefore, riboflavin has been classified as Class II Moderate Risk of Bias in its role as an intervention for migraine in adults according to the American Academy of Neurology evidence‐based rating [18]. However, the effectiveness of riboflavin in paediatric migraine is less clear [15]. The efficacy of riboflavin in paediatric migraine management has been summarised using narrative reviews [7, 11, 15, 19, 20, 21, 22] and two systematic reviews [23, 24], but these were fundamentally limited in their methodology. Hence, the aim of this systematic review is to comprehensively identify and summarise the global evidence on the effectiveness of riboflavin alone and nutraceuticals containing riboflavin in preventing various types of paediatric migraines, including retinal and abdominal migraine. This systematic review strictly adhered to established guidelines for conducting and reporting systematic reviews, ensuring a comprehensive and rigorous synthesis of the available data.

## 2 Methods

This systematic review was conducted and reported adhering to the Joanna Briggs Institute (JBI) methodology for systematic reviews [25] and the Preferred Reporting Items for Systematic Reviews and Meta‐Analyses (PRISMA) [26] and Synthesis Without Meta‐Analyses guidelines [27]. The review protocol was registered with the International Prospective Register of Systematic Reviews (PROSPERO) (Appendix S1) with the designation: CRD42023485443.

### 2.1 Inclusion Criteria

Studies were included if they involved participants below the age of 18 years or where at least 80% of the participants were under 18 years. The intervention of interest was the use of riboflavin or vitamin B2, either alone or as part of a combined therapy, for the prevention of migraine. All dosages and treatment durations were considered. Eligible comparators included any migraine treatment that did not contain riboflavin, different dosages of riboflavin, no treatment or placebo. Outcomes of interest included frequency, such as the number of migraine days or new episodes within a set period; intensity, as measured on scales defined by individual studies; duration, referring to the average length of each episode within a given timeframe; and disability, assessed using tools such as the Paediatric Migraine Disability Assessment Scale (PedMIDAS). Additional outcomes included symptoms like headache, visual aura, vomiting and nausea, as well as any reported side effects.

All quantitative study designs were eligible for inclusion, including randomised controlled trials (RCTs), non‐RCTs, quasi‐experimental studies, observational studies, cohort studies, case–control studies, case reports and case series. Reviews, letters, conference abstracts and opinion pieces were excluded. Studies conducted in any setting and from all regions and countries were considered.

### 2.2 Search Strategy

A preliminary search was conducted on MEDLINE using keywords: riboflavin, migraine and paediatric, to identify subject headings, keywords and index terms to be used in a broader search.

Eight databases and registries were searched from inception until 25 October 2023 to identify published studies. These included: CINAHL, Medline, PubMed, EMBASE, Cochrane Library, Web of Science, Scopus and Clinicaltrials.gov (Appendix S2). EThOS and ProQuest Dissertations and Theses were also searched for grey literature. Reference lists of included studies were screened for further studies. No restrictions on language were imposed.

### 2.3 Study Selection

Retrieved studies from the databases were imported into EndNote (version 20.3) (Clarivate, California, USA), and duplicates were removed. The remaining studies were imported into Rayyan (Rayyan Systems Inc., Massachusetts, USA). Piloting of title and abstract and full‐text screening were conducted independently for 20% of studies by two reviewers (F.A. and E.M.). Discrepancies were discussed, and a third reviewer (J.L.B.) was involved where necessary. A high measure of inter‐rater agreement was achieved for both pilots (> 90%); therefore, further screening was undertaken by one reviewer (F.A.).

### 2.4 Data Extraction

Using a standardised and piloted data extraction form in Excel, data were extracted by one reviewer (F.A.) and double checked by a second reviewer (E.M.). The extraction included: author name, year of publication, title, study location as country and region, context, study design, inclusion criteria, study period, population characteristics such as age and sex, symptoms, intervention, change behaviour, medication during the attacks, comparator, outcome, duration of the intervention, period of measurement, sample size, recruitment method, data collection procedure tool, professionals involved, data analysis, raw data, main results, side effects, study limitations, main conclusion and comments.

### 2.5 Critical Appraisal

Two reviewers (F.A. and E.M.) independently assessed the quality of the studies using the appropriate JBI critical appraisal tools for each study design of the respective paper (RCTs, before‐and‐after studies, case series and case reports [28]). Disagreements were resolved through discussion. Studies were not excluded based on scores because low‐quality studies can also generate important findings [29]. Every question was scored 1 point for yes, 0 for no, 0.5 for unclear or excluded if scored as not applicable (NA). A cumulative score was then calculated. High quality was attributed when all questions were answered as yes; moderate quality when the answers were either yes or unclear; and low quality when at least one answer was no.

### 2.6 Data Synthesis and Analysis

Statistical pooling of quantitative data and meta‐analysis was not possible due to the differences in the interventions, study designs and the methods used. Therefore, a narrative synthesis approach was adopted for summarising the findings. Due to the variation in the interventions and study designs, we grouped studies by the type of migraine (headache migraine vs. others) and the outcome measure. We standardised the definition of the outcome to a common metric, where possible, and reported *p* values as narrated in the included studies. We synthesised the effects for each outcome measure at a comparison level since some studies had more than one intervention group. We used tabulation and vote counting based on the direction of effect, and presented the findings using harvest plots [30]. When multiple measures were reported for different outcomes, these were represented separately in the harvest plots. We indicated the dose of riboflavin and study design in the harvest plots to aid the investigation of heterogeneity.

## 3 Results

### 3.1 Search Results

**FIGURE 1:**
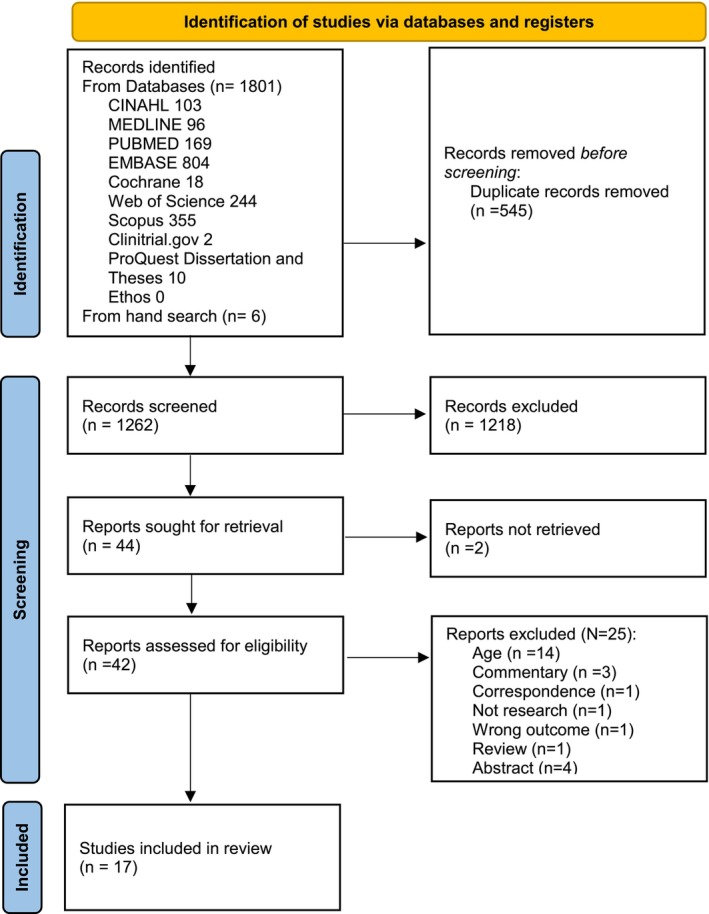
PRISMA flowchart.

The searches yielded 1801 studies from the databases and six further studies from other sources. Following duplicate removal, 1262 studies were screened for title and abstract, with 1218 excluded due to ineligibility. A total of 44 studies underwent full‐text screening, of which two full‐text articles could not be accessed, and 25 studies were excluded due to ineligibility (Appendix S3). The remaining 17 papers met the inclusion criteria and were included in the review (Figure 1).

### 3.2 Characteristics of Included Studies

The 17 included studies were predominantly conducted in Italy [10, 31, 32, 33, 34, 35, 36, 37], with others conducted in Japan [38, 39], the United States [40, 41], Oman [42], Indonesia [43], Australia [44], the Netherlands [45] and Iran [46] (Table 1).

**TABLE 1:** Characteristics of studies included in the review.

Author; year	Study design; country	Sample size; age included (years)	Preventive treatment/comparison; taken with food	Treatment duration	Side effects	Results
Headache migraine						
MacLennan; 2008 [44]	RCT; Australia	48; mean 11.1 ± 2.1	Riboflavin (200 mg/day, *n* = 27)/placebo (200 mg/day orange food dye, *n* = 21); not specified	12 weeks	Riboflavin group: change in urine colour (*n* = 4), frequent tension headaches (*n* = 1); placebo group: diarrhoea 3 days/month and viral symptoms (*n* = 1)	No significant difference between groups for the proportion of patients with 50% or higher reduction in frequency of episodes/month (*p* = 0.125); increase in mean duration in both groups; no change in severity in both groups; mean number of attacks treated with analgesics decreased in both groups; mean number of days with nausea and vomiting decreased in the placebo but not in the riboflavin group
Condo; 2009 [33]	Retrospective before–after; Italy	41;8–18	Riboflavin (200 mg/day *n* = 21 or 400 mg/day *n* = 20)/baseline; yes	3 months (*n* = 40), 4 months (*n* = 14) or 6 months (*n* = 11)	Vomiting (*n* = 1, dropped out), increased appetite (no weight gain, *n* = 1), temporary yellow‐orange urine (*n* = NA)	Significantly reduced attack frequency and intensity after 3 or 4 months (*p* < 0.01), but not at 6 months (*p* > 0.05). 12.5% of patients did not need symptomatic drugs during the treatment; 77.1% found symptomatic treatment more effective during trial. No significant difference between frequency/intensity responders and nonresponders for both 200/400 mg/day doses, migraine types and age of headache onset. Significant prevalence of males in the intensity‐responder group (*p* < 0.05) and < 12 years in the frequency‐responder group (*p* < 0.05). Improvement of symptoms in patients with aura.
Bruijnl; 2010 [45]	RCT; the Netherlands	42; 6–13	Riboflavin (50 mg/day, *n* = 20)/placebo (100 mg of carotene/day, *n* = 22); yes	16 weeks	No side effects	No significant difference in reduction of mean frequency (*p* = 0.44) between groups. Significant reduction of mean frequency of attacks in tension‐type headache in the riboflavin group (*p* = 0.04). No significant difference in change or reduction of mean intensity or mean duration of migraine and TTH attacks between groups.
Athaillah; 2012 [43]	RCT; Indonesia	98; 12–18	Riboflavin (400 mg/day, *n* = 50)/placebo (ingredients not specified, *n* = 48); not specified	3 months	Riboflavin group: polyuria (*n* = 18), diarrhoea (*n* = 12); placebo group: polyuria (*n* = 10), diarrhoea (*n* = 4)	Significant reduction in frequency (1st, 2nd and 3rd month), in duration (2nd and 3rd month, *p* = 0.012 and *p* = 0.01), in fMIDAS score 3rd month *p* = 0.001) compared to placebo
Talebian; 2018 [46]	RCT; Iran	90; 5–12	Riboflavin (100 mg low‐dose or 200 mg/day high‐dose)/placebo (100 mg carotene); yes	12 weeks	No side effects	Significant decrease in number of attacks/month and mean duration in the high‐dose group (*p* = 0.000) but not in the low‐dose group (*p* = 0.49, *p* = 0.69) compared with the placebo. No significant reduction in intensity for both low and high doses compared to placebo
Yamanaka; 2020 [38]	Retrospective before–after, Japan	68; 6–15	Riboflavin (younger patients 10 mg *n* = 13 or older patients 40 mg/day *n* = 55)/baseline; not specified	3 months	No side effects	Significant reduction in median frequency (*p* = 0.000) in both groups. No significant differences in responders and nonresponders and reduction rates between age groups.
Das; 2020 [41]	Retrospective before–after; USA	42; mean 13.38 ± 3.38	Riboflavin (100 or 200 mg/twice daily, weight‐based dosing)/baseline	2–4 months	No side effects	Significantly reduced mean headache days/month (*p* < 0.01), mean intensity (*p* = 0.001) and duration in hrs (*p* < 0.001). Positive correlation between riboflavin and reduced use of acute medication (*p* = 0.05).
Usai; 2010 [10]	Prospective before–after; Italy	24; mean 13.4 ± 2.1	Supplement (Ginkgolide B 80 mg, coenzyme Q10 20 mg, riboflavin 1.6 mg and magnesium 300 mg/twice daily)/baseline; yes	3 months	No side effects	Significant decrease in mean number of days/months (0.0015) and number of analgesics used (*p* = 0.013) compared to baseline.
Usai; 2011 [10]	Prospective before–after; Italy	30; mean 13.5 ± 2.2	Supplement (Ginkgolide B 80 mg, coenzyme Q10 20 mg, riboflavin 1.6 mg and magnesium 300 mg/twice daily)/baseline; yes	3 months	No side effects	Significantly reduced number of attacks/month and the number of analgesics compared to baseline after 3 and 12 months follow‐up (*p* = 0.000)
Esposito; 2011 [35]	Prospective before–after; Italy	119; mean 9.7 ± 1.42	Supplement (Ginkgolide B 80 mg, coenzyme Q10 20 mg, riboflavin 1.6 mg and magnesium 300 mg/twice daily)/baseline; yes	3 months	No side effects	Significant decrease in mean monthly frequency compared to baseline (*p* < 0.01)
Esposito; 2012 [36]	Quasi‐experimental, Italy	374; mean 10.7 ± 1.8	Supplement 1 (*n* = 187, Ginkgolide B 80 mg, coenzyme Q10 20 mg, riboflavin 1.6 mg and magnesium 300 mg/twice daily)/Supplement 2 (*n* = 187, 250 mg L‐tryptophan, 50 mg 5‐hydroxytryptophan from *Griffonia simplicifolia*, 9 mg vitamin PP, 1 mg vitamin B6)/twice daily/baseline; yes	6 months	Supplement 1: *n* = 8 mild and transient nausea or abdominal pain in the 1st week. Supplement 1: *n* = 6, daytime somnolence or vertigo in the 1st week.	Significant reductions in headache frequency compared to baseline (*p* < 0.001) and greater than 50% (clinical significance) for both supplements. This reduction was significantly greater (*p* < 0.001) for Supplement 1 compared to Supplement 2. Headache duration (hrs), pain intensity, PedMIDAS and Behaviour Index were significantly lower in both groups compared to baseline (*p* = 0.001), with no significant differences between the groups at baseline, but significantly lower for Supplement 1 group compared to Supplement 2 (*p* < 0.001).
Moscano; 2019 [32]	Before–after; Italy	91; mean 14.4 ± 2.2	Partena supplement (Mg^2+^ 169 mg, CoQ10 20 mg, riboflavin 4.8 mg, feverfew 150 mg, *Andrographis paniculata* 100 mg)/twice daily/baseline	16 weeks	4.4% diarrhoea and vomiting	Significantly reduced frequency and pain intensity (*p* < 0.001) in patients with or without aura (*n* = 51). Significantly reduced frequency (*p* < 0.001), but no amelioration of pain intensity (*p* > 0.05) in tension‐type headache patients (*n* = 20).
Onofri; 2020 [31]	Quasi‐experimental; Italy	99; 6–17	Compound 1: Mg citrate, Mg oxide and Mg aspartate; Compound 2: bisglycinate Mg along with L‐tryptophan, niacin, riboflavin and vitamin D; and Compound 3: mixture of Mg oxide, Partenium, *Andrographis paniculata* , coenzyme Q10 and riboflavin/compounds or baseline	12 months	No side effects	Significant reduction in Migraine Index (MI) for all compounds (*p* < 0.001) and use of attack therapy compared to baseline. Compounds 2 and 3 are more effective on MI in patients with migraine without aura when compared to Compound 1 (*p* = 0.00089). Compounds 1 and 2 are more effective on MI on migraine with aura (*p* = 0.0044) and on frequent episodic tension‐type headache (*p* = 0.052).
Other migraine types or headache migraine associated with a specific condition						
Carotenuto; 2013 [37]	Before‐after; Italy	18 (neurofibromatosis Type I); mean 8.4 ± 1.65	Supplement (Ginkgolide B 80 mg, coenzyme Q10 20 mg, riboflavin 1.6 mg and magnesium 300 mg/twice daily)/baseline; not specified	6 months	Not reported	Significant decrease in mean frequency/month, mean pain intensity/month, mean duration/month of attacks, PedMIDAS score (*p* < 0.001)
Abouzari; 2019 [40]	Case report; USA	1 (Meniere disease); 5	Riboflavin 100 mg, magnesium 200 mg twice daily/NA	6 weeks, 3 months, 4 months	Not reported	Normal audiogram and resolution of previous symptoms (6 weeks), asymptomatic with no vertigo and hearing maintenance (3 and 4 months)
Al Lawati; 2022 [42]	Retrospective before–after; Oman	43 (Abdominal migraine); mean 6.8 ± 2.6	Riboflavin (R group, 10 mg/Kg/day, max 300 mg/day, *n* = 20); riboflavin + pizotifen (RPI group, 1 mg/day, *n* = 2), riboflavin + propranolol (RPR group, 0.5 mg/kg/twice daily max 10 mg/dose, *n* = 1)/baseline	NA	Not reported	Improvement of abdominal pain in 90% of R group and 100% of RPI group. Significant association of therapy (not specified) with vomiting (*p* = 0.039) and fatigue symptom not significant (*p* = 0.06). Association of R group with vomiting (OR = 14.4, *p* = 0.01)
Morishita; 2022 [39]	Case report; Japan	1 (retinal migraine); 12	Riboflavin 40 mg/NA	3 months	Not reported	Subsided headache, improved Headache Impact Test‐6TM and PedMIDAS scores

The main study design used was an uncontrolled before–after design [10, 32, 33, 34, 35, 37, 38, 41, 42], with other studies using case reports [39, 40], RCTs [43, 44, 45, 46], or quasi‐experimental [31, 35] designs. The sample sizes in the studies ranged from one to 374 participants, with the ages of the recruited individuals spanning from 5 to 18 years. Sixteen studies recruited participants from hospitals [10, 31, 32, 33, 34, 35, 36, 37, 38, 39, 40, 41, 42, 44, 45, 46], with one study also recruiting from schools [44] and the remaining study recruiting from faith centres [43]. Most of the studies included children with headache migraines [10, 31, 32, 33, 34, 35, 36, 38, 41, 43, 44, 45, 46], one study included children with abdominal migraines [42], and one study included children with retinal migraines [39]. Other specific conditions affecting patients with headache migraines were described in two papers: one with neurofibromatosis Type 1 [37], and one with Meniere disease [40]. Doses of riboflavin given as a monotherapy ranged from 10 to 400 mg, with specific doses of 10 mg a day [38], 40 mg a day [38, 39], 50 mg a day [45], 100 mg a day [40, 46] or twice a day [41], 200 mg a day [33, 40, 44, 46], or twice a day [41], and 400 mg a day [33, 43]. One study administered riboflavin (10 mg/kg, max 300 mg/day) either as a monotherapy or as part of a combination therapy with pizotifen (1 mg/kg daily) or propranolol (0.5 mg/kg twice daily, max 10 mg per dose) [42]. In one study, a commercially available supplement *Partena* (NEURAXPHARM, Italy, SpA), containing riboflavin (4.8 mg/tablet) and other ingredients (magnesium, feverfew, *Anrographis paniculata* and CoQ10) [32], was administered to study participants (2 tablets/day for the first 4 weeks and 1 tablet/day for the other 12 weeks). Another study [40] used riboflavin (100 mg twice daily) and magnesium (200 mg twice daily). A further study [31] used three different combined therapies with no dosage specified: Compound 1: magnesium citrate, magnesium oxide and magnesium aspartate; Compound 2: bisglycinate magnesium, L‐tryptophan, niacin, vitamin D and riboflavin; and Compound 3: magnesium oxide, *Partenium*, *Andrographis paniculata* , CoQ10 and riboflavin. The remaining five studies used a combination therapy containing Ginkgolide B 80 mg, CoQ10 20 mg, vitamin B2 1.6 mg and magnesium 300 mg [10, 34, 35, 36, 37]. The duration of treatments varied between 6 weeks and 12 months, and the follow‐up period ranged from 1 to 12 months. In the studies which included control groups, four RCTs had a placebo control group. The comparator group in two of the RCTs was carotene 100 mg [45, 46], another study used an orange food dye 200 mg [44] and the fourth study used an unspecified placebo [43]. One study [31] compared three different compounds and baseline data, with two of the compounds containing riboflavin. One study [36] compared two supplements, with only one of the two containing riboflavin. The remaining 11 studies [10, 32, 33, 34, 35, 37, 38, 39, 40, 41, 42] compared riboflavin treatment with baseline data. Pain relief prescribed to participants to manage attacks included acetaminophen, ibuprofen, nimesulide, ketoprofen, acetylsalicylic acid, metamizole sodium, noramidopyrine, zolmitriptan, sumatriptan, piroxicam and indomethacin.

### 3.3 Methodological Quality

The methodological quality of the studies was varied, ranging from high to low (Appendix S4).

Of the four RCTs, three were of high quality [44, 45, 46], and one of moderate quality [43]. Among the 11 uncontrolled before–after or quasi‐experimental studies, one was high quality [36], one moderate [31] and the remaining nine were low quality [10, 32, 33, 34, 35, 37, 38, 41, 42]. One of the case reports was high quality [40], but the other was low quality [39].

### 3.4 Effectiveness in Headache Migraine Studies

#### 3.4.1 Frequency

**FIGURE 2:**
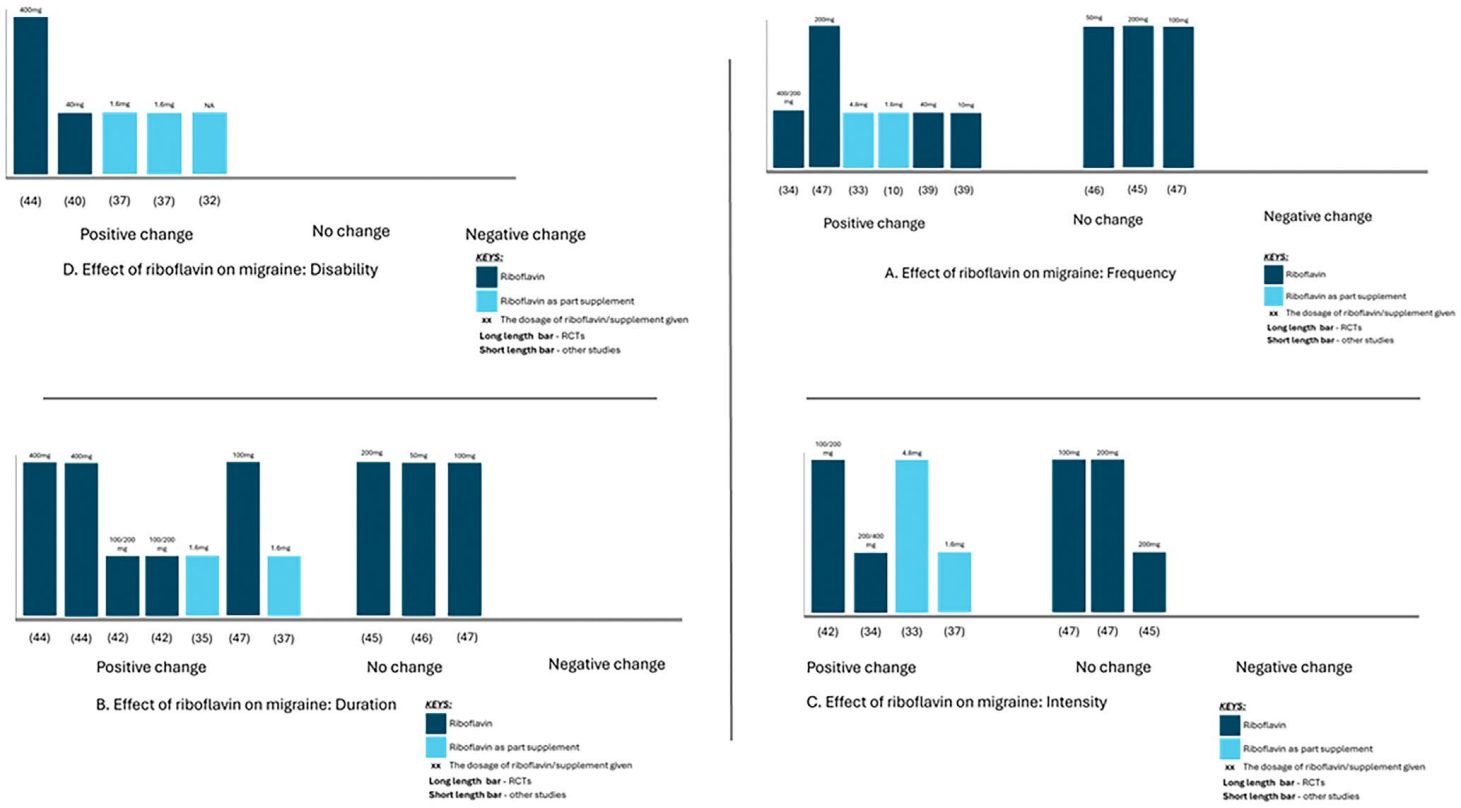
Harvest plot. Effect of riboflavin on migraine in children.

Seven of the included studies (nine comparisons) assessed the effects of riboflavin or combined therapies with riboflavin on the frequency of migraine episodes in a set period, defined as the number of migraine episodes per attack. Six of the nine comparisons demonstrated that these treatments were generally effective in reducing migraine frequency (Figure 2). However, most studies which found a significant reduction in the frequency of migraine did not use an RCT design. Conversely, most studies that did use an RCT design found no significant effect on the frequency of migraine.

Significant reductions in the frequency of attacks of migraines in a month were seen with riboflavin compared to the baseline (*p* < 0.001) [10]. A significant reduction in mean attack frequency was seen with riboflavin (200 or 400 mg/day) compared to baseline (*p* < 0.01) [33]. High‐dose riboflavin (200 mg/day) significantly decreased the number of headache attacks per month compared with placebo (*p* < 0.001). In contrast, no significant effect was seen with low‐dose riboflavin (100 mg/day, *p* = 0.49) compared with placebo [46]. A significant overall reduction in the median frequency of headache episodes compared to baseline was seen at 3 months (*p* = 0.01), with a similar reduction being seen in each riboflavin group (10 and 40 mg/day) [38].

A significant reduction in the number of headache episodes at the end of the 16‐week treatment period was seen with combined therapy with riboflavin (*Partena*) [32]. Conversely, no significant difference was seen between riboflavin (50 mg/day) and placebo in the monthly migraine frequency in the 16 weeks of treatment (*p* = 0.44), except in those with tension‐type headaches (*p* = 0.04) [45].

No significant difference in the number of migraine attacks in 4 weeks was seen between riboflavin (200 mg/day) and placebo following 12 weeks of intervention (*p* = 0.125) [44].

#### 3.4.2 Duration

Two measures of duration were assessed within the included studies: (i) the number of days with a migraine and (ii) the number of hours with a migraine. Seven studies (10 comparisons) assessed this outcome, with most reporting a significant reduction in patients supplemented with riboflavin (Figure 2).

When considering the number of days with a migraine as the measure of duration, of the four studies (four comparisons) assessing this outcome, three reported a significant reduction in patients supplemented with riboflavin.

A significant reduction in mean headache days per month following 2–4 months of riboflavin use (100 or 200 mg twice daily) was found compared with the baseline (*p* < 0.001) [41]. An RCT [43] found that 400 mg/day of riboflavin significantly reduced the mean number of days with migraine/month in the 2nd and 3rd month of treatment compared to placebo (*p* = 0.013, 0.029, respectively). However, the mean number of days with migraine/month was significantly higher in the 1st month of treatment compared to placebo (*p* = 0.010). A significant decrease in the mean number of days with migraine per month was seen using the riboflavin supplement compared to baseline (*p* = 0.0015) [34]. Conversely, no significant difference in the mean number of migraine days between the riboflavin treatment (200 mg/day) and the placebo group was seen following 12 weeks of intervention [44].

Of the five studies (six comparisons) assessing the number of hours during a migraine episode as the measure of duration, four comparisons found a significant reduction in patients supplemented with riboflavin (Figure 2).

Two studies reported a significant reduction in headache duration with riboflavin compared to baseline (*p* < 0.001, *p* = 0.001, respectively) [36, 41]. A RCT [43] found the mean headache duration per month was significantly shorter in the riboflavin group compared to the placebo in the 2nd (*p* = 0.012) and 3rd (*p* = 0.001) months. However, no significant difference was seen between the treatment groups during the 1st month (*p* = 0.404) [43]. High‐dose riboflavin (200 mg) significantly reduced the mean duration of headache attacks compared to placebo (*p* < 0.001) and low‐dose riboflavin (100 mg) (*p* < 0.001) [46]. However, no significant effect on the mean duration of headache attacks was seen for low‐dose (100 mg) riboflavin compared to placebo (*p* = 0.69) [46]. Conversely, riboflavin (50 mg/day) found no significant difference in the duration of each migraine attack (*p* = 0.15) and tension‐type headache attacks (*p*‐value not narrated) compared to placebo [45].

#### 3.4.3 Intensity

Six studies (seven comparisons) reported the effect of riboflavin or combined therapy on the intensity of migraine using different pain scales, with mixed results seen across the studies (Figure 2).

A statistically significant decrease in migraine intensity was reported following 3 months of riboflavin (200 or 400 mg/day) treatment (*p* < 0.01). In contrast, this effect was not maintained in patients who took riboflavin for 4 or 6 months (*p* > 0.05) [33]. Likewise, mean headache intensity was significantly reduced after treatment with riboflavin (100 or 200 mg twice daily; *p* < 0.001) [41]. However, in another study, low‐dose (100 mg/day) and high‐dose (200 mg/day) riboflavin did not significantly reduce migraine intensity compared to placebo (*p* = 0.71, *p* = 0.74, respectively) [46]. Pain intensity was significantly lower following the treatment with riboflavin (*p* < 0.001) [36]. Combined therapy with riboflavin using *Partena* supplement found pain intensity was not significantly different with tension‐type headache (*p* > 0.05) [32]. However, a significant pain reduction was seen in those with migraine with or without aura (*p* < 0.01 and *p* < 0.0001, respectively) [32]. The reduction in pain in those with migraine with or without aura remained significant through to 16 weeks follow‐up after the end of treatment (*p* < 0.001 and p < 0.001, respectively) [32]. No change in migraine severity was seen for those treated with riboflavin (200 mg/day) and control [44].

#### 3.4.4 Disability

Disability due to migraine was assessed as an outcome in five papers using the PedMIDAS score, Migraine Index or Behaviour Index. Positive effects of riboflavin or combined therapy with riboflavin were seen in all comparisons: PedMIDAS score [36, 37, 39, 43], Behaviour Index and [36] Migraine Index [31] (Figure 2).

The mean PedMIDAS score after 3 months of riboflavin treatment was significantly lower compared to placebo (*p* = 0.001) [43]. The use of a supplement containing riboflavin reported a significantly lower PedMIDAS score and a lower Behavioural Index score, compared to baseline (*p* < 0.001) [36]. A case study found that the PedMIDAS score reduced from 15 to 2 following treatment with riboflavin [39]. Combined therapies, which included riboflavin, significantly reduced the Migraine Index score in people with migraine without aura (*p* < 0.001) and migraine with aura (*p* < 0.01) [31]. However, no significant reduction was seen in those with tension‐type frequent headaches (*p* = 0.052) [31].

#### 3.4.5 Effects on the Use of Analgesics

Three studies reported reductions in the use of analgesics during migraine attacks [10, 31, 34] after the use of riboflavin compared to baseline, and after 3‐ and 12‐month follow‐up [10].

#### 3.4.6 Side Effects

Eight studies reported no side effects associated with riboflavin; however, a further five studies reported side effects associated with riboflavin, mainly associated with gastrointestinal symptoms. The remaining four studies did not report this data. Polyuria (36% vs. 20.8%) and diarrhoea (24% vs. 8.4%) were significantly more likely to be reported as side effects in the riboflavin group compared to placebo (*p* = 0.004) [43]. The change in the number of days of vomiting and feeling nauseous was higher in the riboflavin group (35–38 days) compared to the placebo (25–7 days) [44]. In one study, 4.4% of patients interrupted treatment with riboflavin supplement (*Partena*) due to vomiting and nausea [32]. Mild and transient nausea or abdominal pain was reported in eight patients in the 1st week of treatment with riboflavin, and daytime drowsiness or vertigo in six patients [36]. One patient in the riboflavin group reported vomiting, and another patient reported an increase in appetite without weight gain [33]. A change in urine colour was noted in some patients in two studies [33, 44].

### 3.5 Outcomes in Other Conditions With Migraine

Four studies were focused on less frequent migraine or rare conditions where riboflavin was prescribed to prevent migraine episodes.

Riboflavin significantly reduced the mean frequency, mean pain intensity, mean duration and PedMIDAS score compared to baseline (*p* < 0.001) in patients with neurofibromatosis Type I [37].

A case study investigated the efficacy of riboflavin in preventing symptoms of Meniere disease related to migraine headaches [40]. Significant improvements in symptoms were seen with combined therapy of 100 mg riboflavin (with 200 mg twice/day, dietary and lifestyle changes) within 6 weeks [40].

A case study investigated the efficacy of riboflavin for the prevention of retinal migraine [39]. Significant improvements were seen with combined therapy with 40 mg riboflavin, 200 mg ibuprofen, 10 mg domperidone and education on headache management strategies [39]. After 3 months, headaches had reduced significantly, with notable improvement in the Impact Headache Test‐6 (from 76 to 24) and PedMIDAS (from 15 to 2) [39].

Another study in patients with abdominal migraines found that 90% of patients receiving riboflavin (10 mg/kg, max 300 mg/day) improved abdominal pain [42], but increases in vomiting were seen (*p* = 0.01). Improvements in abdominal pain were also seen in two patients who received a combination of riboflavin (10 mg/kg, max 300 mg/day with pizotifen 1 mg/kg daily) [42]. However, no improvement was seen in the single patient who had combined therapy of riboflavin (10 mg/kg, max 300 mg/day) with propranolol (0.5 mg/kg twice daily, max 10 mg per dose) [42]. The lack of improvement was thought to be due to a lack of compliance [42].

## 4 Discussion

This systematic review suggested that, while the evidence was not robust, the included studies showed promising results for the use of riboflavin as a monotherapy or in combination therapy in preventing paediatric migraines.

In addition, two studies, although not meeting the inclusion criteria due to being available only as abstracts [47, 48], supported the findings of this systematic review. The first study found that 400 mg riboflavin was effective in reducing headache days and intensity of migraine in various headache types [47]. The second study found riboflavin given as monotherapy or as a combined therapy to have a significant reduction in the number of headache days and PedMIDAS scores [48]. Notably, no side effects were reported in either of these two studies.

The need for prophylaxis for paediatric migraine is generally determined by the number of attacks per month and their impact. Migraines occurring twice monthly may not need prophylaxis; three to four times may warrant it; and more than five times monthly strongly suggests it [49]. The use of riboflavin treatment is generally considered safe to prescribe by clinicians, and it is not expensive. Few and mild side effects were seen in our review, and in most cases, study authors noted that side effects had multifactorial causes and could not confidently be linked to treatment, such as change in urine colour, vomiting and diarrhoea.

However, some factors may inhibit the efficacy of riboflavin. For instance, two studies [11, 33] identified potential inhibitors such as comorbid headaches, male sex and age under 12 years. Although the evidence for potential inhibitors was insufficient and sometimes contradictory, as highlighted by another study [46].

The variable effectiveness of riboflavin in paediatric patients, compared to adults, may be due to several factors. Insufficient dosing of riboflavin can affect mitochondrial metabolism. The higher metabolic rate in children compared to adults might suggest the need for higher doses to achieve effectiveness. However, the human body can absorb a maximum of 27 mg from a single dose, with absorption saturation occurring at 30–50 mg and decreasing at higher doses. Therefore, administering such high doses once daily might be ineffective given the low maximum absorbance level [23]. Riboflavin's half‐life is between 1 and 2 h, indicating the potential need for multiple daily doses or a sustained‐release product. Although our review found that few studies reported twice‐daily administration, most used a once‐daily regimen. Additionally, the timing and context of administration, such as taking the supplement after meals with a full stomach, were not consistently reported, potentially impacting the effectiveness and the study results.

The strong placebo effect in migraine treatments for children also poses a challenge. Studies should account for a 20% placebo responder rate and a 60% treatment responder rate [50], but sample size calculations in the included trials were insufficient to exclude placebo effects. At this point, paediatricians should take advantage of the evidence available on the riboflavin effect and the placebo effect, trying to expand therapeutic outcomes [51]. The use of analgesic treatments in the included trials was not clearly defined, although some studies noted a reduction in medication use in riboflavin groups.

Regarding the outcomes across our included studies, several discrepancies were noted and pose a challenge in result interpretation. Most studies reported statistically significant results or tendencies when patients underwent riboflavin treatment, while others did not. For example, two studies that assessed the effect of riboflavin on the number of migraine days yielded mixed results. Das et al. [41] reported a significant decrease in mean headache days per month after four to 16 weeks of riboflavin use compared to baseline. In contrast, MacLennan et al. [44] found no significant difference in migraine days between treatment and placebo groups following a 12‐week intervention. One potential explanation for the discrepancy could be the dosing regimen: Das et al. [41] adopted a twice‐daily weight‐based dosing strategy (100 and 200 mg twice a day for children weighing 20–40 kg and greater than 40 kg, respectively). In contrast, MacLennan et al. [44] administered a once daily 200 mg dose, irrespective of weight. Given riboflavin's short half‐life, once‐daily dosing may have been insufficient to produce a significant effect, and body size may have influenced riboflavin responsiveness in the MacLennan et al. study [44]. Another potential explanation is the sample size difference: MacLennan et al. [44] compared 27 individuals in the treatment group with 21 in the placebo group, while Das et al. [41] compared the baseline results of 42 individuals with their outcomes following treatment. The small number of patients in the study of MacLennan et al. [44] precludes the ability to completely exclude a false negative result. A similar study conducted in an adult population found that riboflavin, when administered in high doses (400 mg), was effective in reducing the number of migraine days [52]. Further research into optimal dosing strategies on treatment outcomes is warranted to clarify these findings and optimise riboflavin's efficacy in migraine management.

When assessing the effects of riboflavin supplementation on the frequency of migraine episodes, studies showed that riboflavin is generally effective in reducing it. Varying doses of 50–400 mg of riboflavin were administered over periods ranging from 12 to 24 weeks. One of the studies [33] recommended 16 weeks of riboflavin treatment for optimal response. Although the study found riboflavin did not significantly reduce migraine frequency after 24 weeks of treatment, potentially due to the smaller number of patients reaching this phase [33].

In another study [46], no significant difference was found between low‐dose riboflavin groups (100 mg) and placebo, whereas a notable decrease in migraine frequency was observed in the high‐dose group (200 mg) compared to placebo. This suggests a potential dose‐dependent response of migraines to riboflavin. Furthermore, a study included in this review [45] reinforced this notion, as administration of 50 mg of riboflavin did not result in a significant reduction of migraine frequency compared to the placebo group. Collectively, these findings underscore the importance of further investigating the influence of riboflavin dosage on its efficacy against migraines [53].

Additionally, one study reported no significant reduction in migraine intensity, irrespective of whether participants received high or low doses of riboflavin [46]. Interestingly, one study [33] highlighted the temporal aspect of riboflavin's efficacy, noting that while doses of 200–400 mg were ineffective over a 4‐ to 6‐month treatment period, they exhibited efficacy as early as the 3rd month. Similarly, another study [41] found riboflavin to be effective after a longer duration of treatment, ranging from 2 to 4 months. The last study [32] noted its effectiveness in individuals both with and without aura, but not in those with tension‐type headaches. The reasons behind this variability remain unclear, suggesting that factors beyond dosage and treatment duration may influence riboflavin's effectiveness in migraine management [32].

Clinicians may consider offering nutraceuticals, including riboflavin, based on favourable outcomes and safety profiles, while educating patients and parents to improve lifestyle through behavioural and physical therapies [54]. For example, dietary changes by avoiding foods such as coffee, chocolate, meat and those containing monosodium glutamate were seen to help the included patients alleviate migraine symptoms [43]. Both endogenous and environmental factors are documented to be relevant for controlling migraine symptoms [38]. These improvements can also be reflected in the impact on schooling, where there is a reduction in school days missed among children receiving riboflavin supplementation [41].

This review also noted the effects of supplements or combination therapies where riboflavin was one of the ingredients. It is important to note that the efficacy is more likely to be determined by the synergistic effect of all the ingredients included and their dosages, and not by the riboflavin itself. In the combined therapies included in this review, the dosage of riboflavin was much lower than in studies that used riboflavin as monotherapy. For example, the potential synergy among ingredients like CoQ10, Ginkgolide B, magnesium and feverfew with riboflavin has shown promising results in studies, although the quality of evidence remains low [21, 54, 55]. The five studies included in our review tested a supplement containing these five ingredients in patients only without aura, showing promising effects in preventing migraine [10, 34, 35, 36, 37].

Additionally, this review found positive effects of riboflavin or supplements containing it, when administered in patients with complex conditions such as Meniere disease and neurofibromatosis Type I, or in cases of abdominal migraine and retinal migraine where headache is also present. For example, it has been noted that there is an overlap among diseases like Meniere disease, vestibular migraine and cochlear migraine. These may be variants of each other, implying that therapeutic approaches typically adopted for headache migraines involving lifestyle and dietary changes, as well as certain supplements including riboflavin, could be beneficial across these conditions [40, 56].

### 4.1 Limitations of the Included Studies

This systematic review revealed several limitations of the included studies that must be considered when interpreting the findings.

Firstly, the geographical coverage of the studies included in this review was not comprehensive, potentially limiting the generalisability of the findings to different populations. The variation in study designs is another significant limitation, with a notable need for more RCTs to provide higher‐quality evidence on the efficacy of riboflavin in paediatric migraine prevention. Many of the studies included in the review had small sample sizes, which may affect the reliability and statistical power of the results, without overcoming the placebo effect. Additionally, the studies encompassed a wide range of ages among paediatric participants, introducing variability in how riboflavin affects different age groups. The dosages of riboflavin administered in the studies were not always based on weight or age, leading to inconsistencies in the treatment regimen. This variability could make it challenging for clinicians to determine the optimal dosage for different age groups. The timing of riboflavin administration was not consistently reported across studies. Given riboflavin's short half‐life, the timing and frequency of dosing could significantly impact its effectiveness. Furthermore, not all studies indicated whether riboflavin was administered with food, which could affect its absorption and overall efficacy. The duration of riboflavin treatment also varied among studies, making it difficult to draw definitive conclusions about the optimal length of therapy needed to see beneficial effects. There was a distinction between studies that used riboflavin as a monotherapy and those that used it in combination with other supplements, at lower dosages. The potential synergistic or antagonistic effects of combination therapies need further exploration to understand their efficacy compared to riboflavin monotherapy.

An acknowledged limitation in our review concerns the potential omission of relevant research on the efficacy of supplements containing riboflavin. This limitation arose from the possibility that studies may not explicitly mention riboflavin or vitamin B2 in their abstracts, titles or keywords, having prioritised the investigation of other ingredients within the supplements. To mitigate this issue, we thoroughly screened the reference lists of included studies and consulted previous reviews on the topic.

## 5 Implications

The limitations of the current evidence underscore the necessity for future research to adopt standardised methodologies for new RCTs, larger sample sizes and consistent treatment protocols. Such efforts will help clarify the role of riboflavin in paediatric migraine prevention and establish more definitive guidelines for its use. As suggested in a previous review [15], new studies should aim to distinguish riboflavin deficiency in patients and examine markers of inflammation and oxidative stress. Moreover, it has been hypothesised that a genetic component could vary the response to riboflavin therapy [57].

## 6 Conclusion

This systematic review examined the efficacy of riboflavin, alone and in combination therapy, for managing paediatric migraines. Across diverse study designs, dosages and treatment durations, the findings were mixed. While some studies showed reductions in migraine frequency, duration, intensity and disability, others found no significant effects. These inconsistencies suggest that factors beyond dosage and duration may influence outcomes. Despite limitations in evidence certainty, riboflavin appears to be a safe and potentially effective option. Future research should focus on understanding its mechanisms, optimising dosing strategies and identifying patient characteristics linked to better responses.

## Author Contributions

**Elisa Martello:** conceptualization, methodology, data curation, validation, writing – original draft, writing – review and editing, investigation, supervision. **Fatimah Aiyelabegan:** data curation, validation, methodology, writing – original draft, writing – review and editing. **Jemma Orr:** writing – review and editing, validation. **Emma Wilson:** validation, writing – review and editing, methodology. **Joanne R. Morling:** validation, methodology. **Maria Kinali:** investigation, validation, writing – review and editing, visualization. **Titus Joseph:** writing – review and editing. **Dirontsho Koboto:** writing – review and editing. **Surakshya Dhungana:** writing – review and editing. **Prudence Ikechukwu:** writing – review and editing. **Princella Seripenah:** writing – review and editing. **Heidi Emery:** writing – review and editing, visualization, validation. **James Stewart‐Evans:** writing – review and editing, visualization. **Jo Leonardi‐Bee:** conceptualization, investigation, methodology, software, data curation, formal analysis, supervision, resources, writing – original draft, writing – review and editing, validation.

## Conflicts of Interest

The authors declare no conflicts of interest.

## Supporting information


Appendix S1.

Appendix S2.

Appendix S3.

Appendix S4.
