# Supplementary material for: Systematic Review Suggests Nutraceuticals Containing Vitamin B2 Could Provide an Alternative Treatment for Paediatric Migraines
Source: Acta Paediatr. 2025 May 24;114(10):2443–57. doi: 10.1111/apa.70157 (PMC12420874; doi:10.1111/apa.70157)
Supplement: Supplementary file 1 — Appendix S1. [file APA-114-2443-s004.pdf]

Effectiveness of nutraceuticals containing Riboflavin (Vitamin B2) in paediatric migraine: A systematic review

Citation

Fatimah Aiyelabegan, Jo Leonardi-Bee, Emma Wilson, Joanne Morling, Maria Kinali, Titus Joseph, Dirontsho Koboto, Surakshya Dhungana, Prudence Ikechukwu, Priscilla Seripenah, Heidi Emery, Elisa Martello. Effectiveness of nutraceuticals containing Riboflavin (Vitamin B2) in paediatric migraine: A systematic review. PROSPERO 2023 CRD42023485443 Available from: https://www.crd.york.ac.uk/prospero/display_record.php?ID=CRD42023485443

Review question

What is the current scientific evidence on the efficacy of riboflavin (Vitamin B2) or supplements containing riboflavin as preventative treatment for paediatric migraine patients?

Searches

The following bibliographic databases will be systematically and methodically searched (from date of inception) for this review.

• CINAHL

• MEDLINE

• PubMed

• EMBASE

• Cochrane

• Web of Science

• Scopus

• Clinitrial.gov

• Ethos

• ProQuest Dissertation and Theses

Types of study to be included

All quantitative study designs will be included in this review (randomised and non-randomised controlled trials, quasi-experimental studies, observational studies, cohort studies, case-control studies, case reports)

Condition or domain being studied

Migraines are a debilitating neurological disorder characterised by recurrent, severe headaches often accompanied by other symptoms such as nausea, vomiting, and sensitivity to light and sound. Migraines can affect individuals of all age groups, and are frequent among paediatric patients, significantly impacting their quality of life and overall well-being.

The burden of migraines in children and adolescents extends beyond the physical symptoms, encompassing academic performance, social activities, and emotional well-being. Finding effective and safe preventative treatment options for paediatric migraine sufferers is crucial, as it can significantly enhance their quality of life.

Riboflavin, also known as Vitamin B2, is an essential water-soluble vitamin that plays a crucial role in various metabolic processes. It is also known for its potential therapeutic benefits in migraine management. Previous research in adults has indicated that riboflavin supplementation may reduce the frequency and intensity of migraines. However, limited studies have focused on the use of riboflavin-containing nutraceuticals specifically in paediatric populations. The efficacy of riboflavin in paediatric migraine management need to be systematically assessed to provide evidence-based recommendations for clinicians and parents.

Participants/population

Participants below the age of 18 years or where at least 80% of the participants are below the age 18 years

Intervention(s), exposure(s)

Use of riboflavin (vitamin B2) alone or as part of supplements for the prevention of migraine; Any dosage, any treatment duration

Comparator(s)/control

Any treatment for migraine which does not contain riboflavin (vitamin B2); No treatment; Placebo;

Context

All settings are eligible including but not limited to hospitals, communities. Also, all regions and countries are eligible for inclusion into the study

Main outcome(s)

Effects of riboflavin or nutraceuticals containing riboflavin on the number of migraine days in a year, frequency of migraine episodes in a year, migraine intensity (on a scale as defined by individual studies), number of migraine symptoms including but not limited to headache, visual aura, vomiting and nausea

Additional outcome(s)

Duration of the migraine episode, side effects

Data extraction (selection and coding)

Retrieved studies from the databases will be imported into EndNote-20 and duplicate citations will be removed. The remaining studies will be imported into Rayyan. This would be followed by title and abstract screening of 20% of these studies conducted by two reviewers independently. Discrepancies will be discussed between them. In situations of non-agreement, a third reviewer will be involved. If the agreement is greater than 90%, a single reviewer will complete the title and abstract screening. This will be followed by accessing the full text of potentially relevant studies via online search or inter-library loan (ILL). 20% of the full text will be screened by two reviewers independently. Discrepancies will be discussed between them. In situations of non-agreement, a third reviewer will be involved. If the agreement is greater than 90%, a single reviewer will complete the full-text screening.

Using a standardised and piloted data extraction form (Excel), 20% of the data from the included studies data will be double-extracted by two independent reviewers. If the agreement is greater than 90%, a single reviewer will complete the data extraction with a second check by the second reviewer. Disagreements will be discussed among reviewers with the involvement of a third reviewer if consensus is not reached.

Data to be extracted include:

• Study characteristics: Citation details, Study design, Study title, Study period, Country, Context, Inclusion criteria, Exclusion criteria, Population characteristics, Symptoms, Intervention and Comparator, duration of intervention, measurement period, Sample size, Recruitment method, Data collection procedure and tools

• Result data: Outcome data, statistical analysis and effect sizes, Raw data, Main results, Side effect, Study limitations, Main conclusion.

Risk of bias (quality) assessment

The validity and methodological quality of the included studies will be assessed using the JBI critical appraisal tools according to the study design. 20% of the included studies will be evaluated by two reviewers independently. Discrepancies will be discussed between them. In situations of non-agreement, a third reviewer will be involved. If the agreement is greater than 90%, a single reviewer will complete the evaluation.

Strategy for data synthesis

We will initially use a narrative synthesis approach to look systematically at the data and to describe each study based on the review question. If possible, a random-effects meta-analysis will be conducted using the Review Manager 5. This is based on the assumption that the treatment effect of riboflavin varies across studies.

If possible, Cochrane GRADEpro (Grading of Recommendations, Assessment, Development, and Evaluation) software will be used, the primary reviewer will evaluate evidence certainty in meta-analysis. The degree of certainty of the evidence will be ranked as high, moderate, low, or very low.

Analysis of subgroups or subsets

We will assess the heterogeneity by visually observing the forest plot and using the I^2 to quantify statistical heterogeneity. If there is available data, reasons for heterogeneity will be assessed by conducting a subgroup analysis and sensitivity analysis, primarily focusing on the length of time/dosage needed to see the effect of riboflavin in a patient (time-dependent or dose-dependent effect).

Where possible, we will also conduct subgroup or sensitivity analysis based on risk of bias, age group, sex, outcome type, and treatment options (treatment with only riboflavin or in combination with other treatment options). If there is a difference in subgroups, the type of interaction will also be clearly defined.

Also, a publication bias will be assessed using a funnel plot.

Contact details for further information

Dr. Elisa Martello

Elisa.Martello1@nottingham.ac.uk

Organisational affiliation of the review

University of Nottingham, UK

Review team members and their organisational affiliations

Miss Fatimah Aiyelabegan. University of Nottingham, UK

Professor Jo Leonardi-Bee. University of Nottingham, UK

Professor Emma Wilson. University of Nottingham, UK

Assistant/Associate Professor Joanne Morling. University of Nottingham, UK

Dr Maria Kinali. Imperial College London, UK and Portland Hospital-HCA International, UK

Titus Joseph. University of Nottingham, UK

Dirontsho Koboto. University of Nottingham, UK

Surakshya Dhungana. University of Nottingham, UK

Prudence Ikechukwu. University of Nottingham, UK

Priscilla Seripenah. University of Nottingham, UK

Heidi Emery. University of Nottingham, UK

Dr Elisa Martello. University of Nottingham, UK

Type and method of review

Intervention, Meta-analysis, Narrative synthesis, Systematic review

Anticipated or actual start date

10 October 2023

Anticipated completion date

10 February 2024

Funding sources/sponsors

Internal funding from the University of Nottingham

Conflicts of interest

Language

English

Country

England

Stage of review

Review Ongoing

Subject index terms status

Subject indexing assigned by CRD

Subject index terms

MeSH headings have not been applied to this record

Date of registration in PROSPERO

28 November 2023

Date of first submission

27 November 2023

Stage of review at time of this submission

Stage	Started	Completed
Preliminary searches	Yes	No
Piloting of the study selection process	Yes	No
Formal screening of search results against eligibility criteria	No	No
Data extraction	No	No
Risk of bias (quality) assessment	No	No
Data analysis	No	No

The record owner confirms that the information they have supplied for this submission is accurate and complete and they understand that deliberate provision of inaccurate information or omission of data may be construed as scientific misconduct.

The record owner confirms that they will update the status of the review when it is completed and will add publication details in due course.

Versions

28 November 2023
