# Supplementary material for: Systematic Review Suggests Nutraceuticals Containing Vitamin B2 Could Provide an Alternative Treatment for Paediatric Migraines
Source: Acta Paediatr. 2025 May 24;114(10):2443–57. doi: 10.1111/apa.70157 (PMC12420874; doi:10.1111/apa.70157)
Supplement: Supplementary file 2 — Appendix S2. [file APA-114-2443-s001.docx]

**Supplementary S2**

***Search strategy.***

***CINAHL***

Search run: Inception to October 25, 2023; 103 search results

| **#** | **Query** | **Results** |
| --- | --- | --- |
| S1 | (MH "Migraine") | 15,898 |
| S2 | (MH "Headache") | 16,420 |
| S3 | migraine* | 20,847 |
| S4 | S1 OR S2 OR S3 | 33,484 |
| S5 | (MH "Riboflavin") | 1,068 |
| S6 | vitamin b2 | 786 |
| S7 | S5 OR S6 | 1,357 |
| S8 | S4 AND S7 | 103 |

***MEDLINE***

Search run: 1946 to October 24, 2023; 96 search results

1 Migraine Disorders/ 29852

2 Headache/ 31745

3 migraine*.mp. [mp=title, book title, abstract, original title, name of substance word, subject heading word, floating sub-heading word, keyword heading word, organism supplementary concept word, protocol supplementary concept word, rare disease supplementary concept word, unique identifier, synonyms, population supplementary concept word, anatomy supplementary concept word] 47297

4 headache*.mp. [mp=title, book title, abstract, original title, name of substance word, subject heading word, floating sub-heading word, keyword heading word, organism supplementary concept word, protocol supplementary concept word, rare disease supplementary concept word, unique identifier, synonyms, population supplementary concept word, anatomy supplementary concept word] 114000

5 1 or 2 or 3 or 4 138007

6 Riboflavin/ 9495

7 vitamin b2.mp. [mp=title, book title, abstract, original title, name of substance word, subject heading word, floating sub-heading word, keyword heading word, organism supplementary concept word, protocol supplementary concept word, rare disease supplementary concept word, unique identifier, synonyms, population supplementary concept word, anatomy supplementary concept word] 2141

8 6 or 7 10290

9 5 and 8 96

***PUBMED***

Search run: Inception to October 25, 2023; 169 search results

((((migraine[MeSH Terms]) OR (headache[MeSH Terms])) OR (migraine*)) OR (headache*)) AND (((Riboflavin[MeSH Terms]) ) OR (vitamin b2))

***EMBASE***

Search run: Inception to October 24, 2023; 805 search results

1 migraine/ 67407

2 headache/ 276202

3 migraine*.mp. [mp=title, abstract, heading word, drug trade name, original title, device manufacturer, drug manufacturer, device trade name, keyword heading word, floating subheading word, candidate term word] 87844

4 1 or 2 or 3 336601

5 riboflavin/ 19080

6 vitamin b2.mp. [mp=title, abstract, heading word, drug trade name, original title, device manufacturer, drug manufacturer, device trade name, keyword heading word, floating subheading word, candidate term word] 2024

7 5 or 6 19585

8 4 and 7 805

**Cochrane**

Search run: Inception to October 25, 2023; 18 search results

ID Search Hits

#1 MeSH descriptor: [Migraine Disorders] explode all trees 3542

#2 MeSH descriptor: [Headache] explode all trees 5923

#3 (migrain*) (Word variations have been searched) 9939

#4 #1 or #2 or #3 14921

#5 MeSH descriptor: [Riboflavin] explode all trees 497

#6 ("vitamin B2") (Word variations have been searched) 206

#7 #5 or #6 677

#8 #4 AND #7 18

***Web of science***

1: ALL=(migraine disorders) Results: 13727

2: ALL=(headache) Results: 117518

3: ALL=(migraine*) Results: 60745

4: #1 OR #2 OR #3 Results: 140761

5: ALL=(Riboflavin) Results: 16540

6: ALL=(vitamin b2) Results: 2190

7: #6 OR #5 Results: 18113

8: #4 AND #7 Results: 244

**Scopus**

Search run: Inception to October 25, 2023; 355 search results

(ALL ( migraine OR headache ) AND ALL ( riboflavin OR vitamin AND b2 ) )

**Clinicaltrials.gov**

Search run: Inception to October 24, 2023; 2 search results

Condition – Migraine

Other terms - migraine disorders OR migraineurs OR headache

Intervention – Vitamin B2

**Proquest Dissertation and Theses**

Search run: Inception to October 24, 2023; 10 search results

1: ((((((((TS=(migraine disorders)) OR TS=(Migraine) OR TS=(migraineurs)) OR TS=(headache)) Results: 3272

2: (((TS=(Riboflavin)) OR TS=(vitamin B2)) AND TS=(Micro nutrients)) OR TS=(Dietary supplement* Results: 6301

3: #1 AND #2 Results: 10

**Ethos**

Search run: Inception to October 25, 2023; 0 search results

1. Migraine or headache
2. Riboflavin or Vitamin b2
