# Supplementary material for: Systematic Review Suggests Nutraceuticals Containing Vitamin B2 Could Provide an Alternative Treatment for Paediatric Migraines
Source: Acta Paediatr. 2025 May 24;114(10):2443–57. doi: 10.1111/apa.70157 (PMC12420874; doi:10.1111/apa.70157)
Supplement: Supplementary file 3 — Appendix S3. [file APA-114-2443-s003.docx]

Supplementary S3

Excluded papers by full text with reason for exclusion.

| Author | Title | Reason for exclusion |
| --- | --- | --- |
| 1. Boehnke_2004 | High-dose riboflavin treatment is efficacious in migraine prophylaxis: An open study in a tertiary care centre | Age |
| 1. Bougea_2019 | Benefit of coenzyme Q10, riboflavin, petasites and relaxation in the migraine prophylaxis: A Randomized Controlled trial | Age |
| 1. Breen_2003 | High-dose riboflavin for prophylaxis of migraine | Age |
| 1. Chwolka_2023 | Comorbidity or combination - more evidence for cluster-migraine? | Age |
| 1. Schoenen_1998 | Effectiveness of high-dose riboflavin in migraine prophylaxis. A randomized controlled trial | Age |
| 1. Smith_1946 | The role of riboflavin in migraine | Age |
| 1. Vikelis_2021 | Open label prospective experience of supplementation with a fixed combination of magnesium, vitamin b2, feverfew, andrographis paniculata and coenzyme q10 for episodic migraine prophylaxis | Age |
| 1. Hamad_2023 | An open label comparative prospective study of proprietary formula migradep®) versus propranolol for chronic prophylaxis migraine | Age |
| 1. Maizels_2004 | A Combination of Riboflavin, Magnesium, and Feverfew for Migraine Prophylaxis: A Randomized Trial | Age |
| 1. Nambiar_2011 | Oral riboflavin versus oral propranolol in migraine prophylaxis: an open label randomized controlled trial | Age |
| 1. Nct_2017 | Magnesium, Partenium, Andrographis, Co-enzyme Q10 and Riboflavin (PACR) in Migraine Prophylaxis | Age |
| 1. Peng_2022 | Association between the prognostic nutritional index and severe headache or migraine: a population-based study | Age |
| 1. Sandor_2000 | Prophylactic treatment of migraine with β-blockers and riboflavin:: Differential effects on the intensity dependence of auditory evoked cortical potentials | Age |
| 1. Schoenen_1994 | High-dose riboflavin as a prophylactic treatment of migraine: results of an open pilot study | Age |
| 1. Davis_2004 | High-dose riboflavin for the prevention of migraine: can we afford to ignore it? | Correspondence |
| 1. Yee_1999 | Effectiveness of high-dose riboflavin in migraine prophylaxis | Commentary |
| 1. Maizels_2005 | Riboflavin/magnesium/Tanacetum parthenium (feverfew) combination for migraine prophylaxis: Not effective? | Commentary |
| 1. Montagna_1994 | High-dose riboflavin as a prophylactic treatment | Commentary |
| 1. O'Brien_2010 | Vitamins and paediatric migraine: Riboflavin as a preventative medicatio | Review |
| 1. Sabo_2009 | Riboflavin status in 12 pediatric migraine patients is analyzed using the erythrocyte glutathione reductase level activation test (EGR-A) | Wrong outcome |
| 1. Patniyot_2021 | Analysis of nutraceutical medications for migraine prevention in a pediatric and adolescent headache clinic population | Abstract only |
| 1. Saini_2023 | Migraine Disability Evaluation in Indian Children by Pediatric Migraine Disability Assessment (PedMIDAS) Scale: A Prospective Observational Study | Abstract Only |
| 1. Schoenen_1997 | High-dose riboflavin is effective in migraine prophylaxis: Results from a double blind, randomized, placebo controlled trial | Abstract Only |
| 1. Schoenen_1997 | The effectiveness of high-dose riboflavin in migraine prophylaxis: Results from a randomised controlled trial | Abstract Only |
| 1. Anon_2009 | Preventative therapy plays an important role in managing chronic daily headache in paediatric patients | Not research |
