# Supplementary material for: Systematic Review Suggests Nutraceuticals Containing Vitamin B2 Could Provide an Alternative Treatment for Paediatric Migraines
Source: Acta Paediatr. 2025 May 24;114(10):2443–57. doi: 10.1111/apa.70157 (PMC12420874; doi:10.1111/apa.70157)
Supplement: Supplementary file 4 — Appendix S4. [file APA-114-2443-s002.docx]

**Supplement S4. Critical appraisal of included studies.**

**Table S1:** Critical appraisals result of included RCT studies- using the JBI tool (13 questions). Every question received a score based on whether it was answered as 'yes' (1 point), 'no' (0 points), 'unclear' (0.5 points), or 'not applicable (NA)'. A cumulative score was then calculated, excluding 'NA' responses.

| **Questions** | **Author, Year** | | | |
| --- | --- | --- | --- | --- |
|  | Talebian,2018 | Bruijn, 2010 | MacLennan, 2008 | Athaillah, 2012 |
| Was true randomization used for assignment of participants to treatment groups? | Y | Y | Y | Y |
| Was allocation to treatment groups concealed? | Y | Y | Y | U |
| Were treatment groups similar at the baseline? | Y | Y | Y | Y |
| Were participants blind to treatment assignment? | Y | Y | Y | Y |
| Were those delivering the treatment blind to treatment assignment? | Y | Y | Y | Y |
| Were treatment groups treated identically other than the intervention of interest? | Y | Y | Y | Y |
| Were outcome assessors blind to treatment assignment? | Y | Y | Y | U |
| Were outcomes measured in the same way for treatment groups? | Y | Y | Y | Y |
| Were outcomes measured in a reliable way | Y | Y | Y | Y |
| Was follow up complete and if not, were differences between groups in terms of their follow up adequately described and analysed? | Y | Y | Y | Y |
| Were participants analysed in the groups to which they were randomized? | Y | Y | Y | Y |
| Was appropriate statistical analysis used? | Y | Y | Y | Y |
| Was the trial design appropriate and any deviations from the standard RCT design (individual randomization, parallel groups) accounted for in the conduct and analysis of the trial? | Y | Y | Y | Y |
| **Total** | 13 | 13 | 13 | 12 |

**Table 2**: Critical appraisals result of included before – after or quasi-experimental studies - using the JBI tool (9 questions). Every question received a score based on whether it was answered as 'yes' (1 point), 'no' (0 points), 'unclear' (0.5 points), or 'not applicable (NA)'. A cumulative score was then calculated, excluding 'NA' responses

| **Questions** | **Author, Year** | | | | | | | | | | |
| --- | --- | --- | --- | --- | --- | --- | --- | --- | --- | --- | --- |
|  | Al Lawati, 2022 | Condo, 2009 | Das, 2020 | Moscano, 2019 | Yamanaka, 2020 | Usai, 2010 | Usai, 2011 | Esposito, 2011 | Esposito, 2012 | Onofri, 2020 | Carotenuto, 2013 |
| Is it clear in the study what is the ‘cause’ and what is the ‘effect’ | Y | Y | Y | Y | Y | Y | Y | Y | Y | Y | Y |
| Were the participants included in any comparisons similar? | Y | Y | Y | Y | Y | Y | Y | Y | Y | Y | Y |
| Were the participants included in any comparisons receiving similar treatment/care, other than the exposure or intervention of interest? | Y | Y | Y | Y | Y | Y | Y | Y | Y | Y | Y |
| Was there a control group? | N | N | N | N | N | N | N | N | Y | Y | N |
| Were there multiple measurements of the outcome both pre and post the intervention/exposure? | Y | Y | Y | Y | Y | Y | Y | N | Y | Y | Y |
| Was follow up complete and if not, were differences between groups in terms of their follow up adequately described and analyzed? | U | U | N | U | Y | Y | N | Y | Y | U | Y |
| Were the outcomes of participants included in any comparisons measured in the same way? | Y | Y | Y | Y | Y | Y | Y | Y | Y | Y | Y |
| Were outcomes measured in a reliable way? | Y | Y | Y | Y | Y | Y | Y | Y | Y | Y | Y |
| Was appropriate statistical analysis used? | Y | Y | Y | Y | Y | Y | Y | Y | Y | Y | Y |
| **Total** | 7.5 | 7.5 | 7 | 7.5 | 8 | 8 | 7 | 7 | 9 | 8.5 | 8 |

**Table 3**: Critical appraisal result of included case reports studies- using the JBI tool (8 questions). Every question received a score based on whether it was answered as 'yes' (1 point), 'no' (0 points), 'unclear' (0.5 points), or 'not applicable (NA)'. A cumulative score was then calculated, excluding 'NA' responses

| **Questions** | **Author, Year** | |
| --- | --- | --- |
|  | Morishita, 2022 | Abouzari, 2019 |
| Were patient’s demographic characteristics clearly described? | Y | Y |
| Was the patient’s history clearly described and presented as a timeline? | Y | Y |
| Was the current clinical condition of the patient on presentation clearly described? | Y | Y |
| Were diagnostic tests or assessment methods and the results clearly described? | Y | Y |
| Was the intervention(s) or treatment procedure(s) clearly described? | Y | Y |
| Was the post-intervention clinical condition clearly described? | N | Y |
| Were adverse events (harms) or unanticipated events identified and described? | N | Y |
| Does the case report provide takeaway lessons? | Y | Y |
| **Total** | 6 | 8 |
